# Auto-Segmentation Ultrasound-Based Radiomics Technology to Stratify Patient With Diabetic Kidney Disease: A Multi-Center Retrospective Study

**DOI:** 10.3389/fonc.2022.876967

**Published:** 2022-07-04

**Authors:** Jifan Chen, Peile Jin, Yue Song, Liting Feng, Jiayue Lu, Hongjian Chen, Lei Xin, Fuqiang Qiu, Zhang Cong, Jiaxin Shen, Yanan Zhao, Wen Xu, Chenxi Cai, Yan Zhou, Jinfeng Yang, Chao Zhang, Qin Chen, Xiang Jing, Pintong Huang

**Affiliations:** ^1^ Department of Ultrasound in Medicine, The Second Affiliated Hospital of Zhejiang University School of Medicine, Zhejiang University, Hangzhou, China; ^2^ Ultrasound in Medicine and Biomedical Engineering Research Center, The Second Affiliated Hospital of Zhejiang University School of Medicine, Zhejiang University, Hangzhou, China; ^3^ Department of Ultrasound, Sichuan Provincial People’s Hospital, University of Electronic Science and Technology of China, Chengdu, China; ^4^ Department of Clinical Laboratory, Second Affiliated Hospital, Zhejiang University School of Medicine, Hangzhou, China; ^5^ Post-Doctoral Research Center, Hangzhou Supor South Ocean Pharmaceutical Co., Ltd, Hangzhou, China; ^6^ Department of Ultrasound, The People’s Hospital of Yinshang, Anhui, China; ^7^ Department of Ultrasound, Tianjin Third Central Hospital, Tianjin, China; ^8^ Research Center for Life Science and Human Health, Binjiang Institute of Zhejiang University, Hangzhou, China

**Keywords:** ultrasound, radiomics, deep learning, diabetic kidney disease, multicenter

## Abstract

**Background:**

An increasing proportion of patients with diabetic kidney disease (DKD) has been observed among incident hemodialysis patients in large cities, which is consistent with the continuous growth of diabetes in the past 20 years.

**Purpose:**

In this multicenter retrospective study, we developed a deep learning (DL)-based automatic segmentation and radiomics technology to stratify patients with DKD and evaluate the possibility of clinical application across centers.

**Materials and Methods:**

The research participants were enrolled retrospectively and separated into three parts: training, validation, and independent test datasets for further analysis. DeepLabV3+ network, PyRadiomics package, and least absolute shrinkage and selection operator were used for segmentation, extraction of radiomics variables, and regression, respectively.

**Results:**

A total of 499 patients from three centers were enrolled in this study including 246 patients with type II diabetes mellitus (T2DM) and 253 patients with DKD. The mean intersection-over-union (Miou) and mean pixel accuracy (mPA) of automatic segmentation of the data from the three medical centers were 0.812 ± 0.003, 0.781 ± 0.009, 0.805 ± 0.020 and 0.890 ± 0.004, 0.870 ± 0.002, 0.893 ± 0.007, respectively. The variables from the renal parenchyma and sinus provided different information for the diagnosis and follow-up of DKD. The area under the curve (AUC) of the radiomics model for differentiating between DKD and T2DM patients was 0.674 ± 0.074 and for differentiating between the high and low stages of DKD was 0.803 ± 0.037.

**Conclusion:**

In this study, we developed a DL-based automatic segmentation, radiomics technology to stratify patients with DKD. The DL technology was proposed to achieve fast and accurate anatomical-level segmentation in the kidney, and an ultrasound-based radiomics model can achieve high diagnostic performance in the diagnosis and follow-up of patients with DKD.

## Introduction

Diabetic kidney disease (DKD) is a common microvascular complication in patients with diabetes and is the primary cause of kidney failure in ∼40% of diabetic patients (1, 2). In China, an increasing proportion of patients with DKD has been observed among incident hemodialysis patients in large cities, which is consistent with the continuous growth in diabetic patients in the past 20 years (3). DKD diagnosis is based on estimated glomerular filtration rate (eGFR), urinary abnormalities such as proteinuria and microhematuria, and kidney biopsy, which is often avoided in the early stages of DKD.

In patients with suspected kidney function injury, ultrasound imaging is the first imaging technique to be performed for the diagnosis and follow-up of its progression (4). Researchers have demonstrated that certain parameters such as cortical echogenicity and thickness in B-mode ultrasonography, resistance index in color Doppler sonography (4, 5), elastography scores (6) and time-intensity curve parameters in contrast-enhanced ultrasound imaging (7) can effectively reflect the kidney function in patients with chronic kidney disease (CKD). Ultrasound is frequently applied as an available and noninvasive technology for the diagnosis and follow-up of DKD in patients suffering from type II diabetes mellitus (T2DM) for a long duration. However, a conventional ultrasound examination is limited owing to the visual grayscale image, which reduces its potential for identifying a large amount of valuable information. Furthermore, the interpretation of ultrasound images is variable and unreliable owing to inexperienced sonographers, especially in the diffused form of the disease.

Radiomics is a rapidly growing discipline based on quantitative image analysis that reflects image textures and morphology using gray values, which provides a quantitative, solid, and objective foundation for analytic standardization to inform clinical decisions (8). In radiomics technology, a delineated region of interest (ROI) is vital for extracting radiomics variables; however, the accurate anatomical segmentation of ROIs is a time-consuming and experience-dependent process. Recent advances in image segmentation, classification, and registration through deep learning (DL) have considerably expanded the scope and scale of medical image analysis (9). A state-of-the-art network “DeepLabV3+” was reported for semantic image segmentation, which achieved high accuracy when compared to other networks (10).

Therefore, this multicenter retrospective study aimed to achieve an automatic anatomical-level segmentation of the kidney in T2DM patients with/without DKD and to build an ultrasound-image-based radiomics model for diagnosis and follow-up of patients with different stages of DKD. This method can extensively utilize the information contained in conventional ultrasound images and achieve acceptable accuracy, resulting in a quick process that uses easily available resources and demonstrates the potential for further clinical use.

## Materials and Methods

### Study Design and Patients

This study was registered at ClinicalTrials.gov No. NCT05025540 and the informed consent requirement was waived due to the retrospective study design. This multicenter retrospective study was approved by the ethics consultant committee of the Second Affiliated Hospital of Zhejiang University School of Medicine (SAHZU), the People’s Hospital of Yingshang (PHYS) and Tianjin Third Central Hospital (TJTCH), and 162 DKD and 131 T2DM patients were consecutively enrolled in this study between January 2016 and December 2020 and January 2018 and December 2020, respectively, as the control group from SAHZU. Moreover, 35 DKD and 52 T2DM patients from the People’s Hospital of Yingshang (PHYS) and 56 DKD and 63 T2DM patients from Tianjin Third Central Hospital (TJTCH) were also enrolled consecutively.

The following parameters were used to define DKD: 1) urinary albumin-to-creatinine ratio (UACR) > 30 mg/24 h, with an increase greater than twice the original value in three subsequent examinations conducted over 3-6 months; 2) eGFR < 60 ml min^-1^ for more than three months; 3) pathological result of kidney biopsy shows evidence of DKD.

### Clinical Stage of Diabetic Kidney Disease (DKD)

The clinical stage of DKD was defined based on the Chinese guidelines for the diagnosis and treatment of DKD (11, 12). DKD stage I, called as high-filtration stage, was defined as having normal or a marginally elevated eGFR (> 90 mL/min/1.73 m^2^) and negative microalbuminuria; DKD stage II, called as microalbumin stage, was defined as having a urinary albumin excretion rate (UAER) of approximately 20-200 μg/min or 30-300 mg/24 h and eGFR > 60 mL/min/1.73 m^2^; DKD stage III, called as massive albuminuria stage, was defined as having normal UACR >300 mg/g, UAER > 200 ug/min or >300 mg/24 h and eGFR > 15 mL/min/1.73 m^2^; DKD stage IV, called as renal failure stage, was defined as having eGFR < 15 mL/min/1.73 m^2^. In this study, we defined the low DKD stage as the stage lower than stage II and high DKD stage as the stage higher than stage III.

The low-stage DKD was defined as the DKD stage that is lower than stage III; moreover, high-stage DKD was defined as the DKD stage that is higher than or equal to stage III.

### Kidney Ultrasound Scan

A 3-5 MHz convex probe was used for the adult kidney scans. For the kidney, patients with left/right lateral or dorsal positions were scanned in the coronal plane, and B-mode images were recorded. The maximum longitudinal kidney images with the renal sinus and parenchyma were saved. Both sides of the kidney were recorded per patient.

### Inclusive and Exclusive Criteria

#### Inclusive Criteria

Patients were enrolled according to the following criteria: 1) patients with a clinical diagnosis of T2DM and DKD; 2) patients with clear B-mode ultrasound images on both sides of the kidney (left and right); 3) there were no missing values of the selected clinical data such as eGFR and UACR in the electronic medical records.

### Exclusive Criteria

The following criteria were considered for excluding patients: 1) patients with large kidney-space-occupying diseases, such as kidney renal cysts and tumors; 2) ultrasound images with severe shadows or incomplete kidney borders.

### Data Extraction and Model Building

First, we manually and automatically delineated the ROI of the renal parenchyma and sinus.

**Figure 1:**
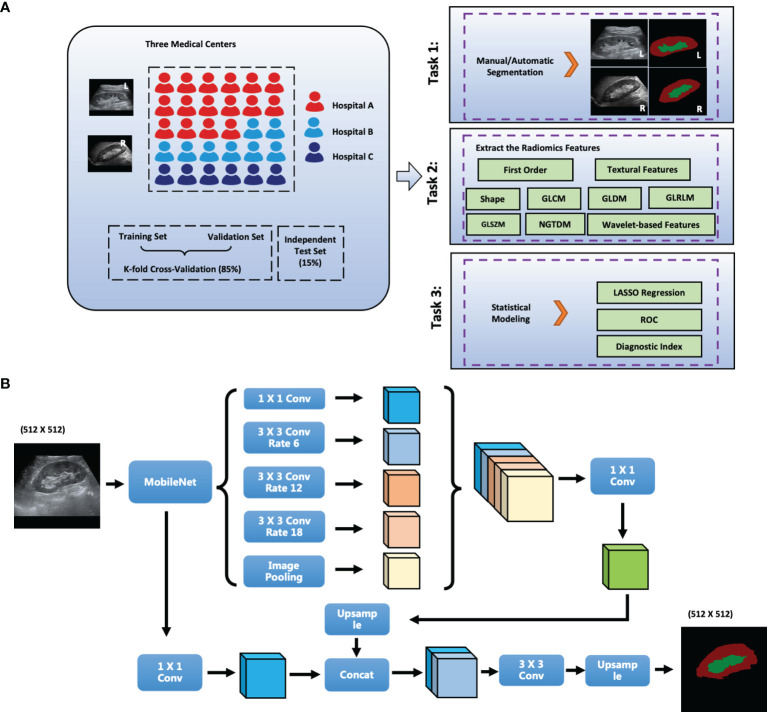
The scheme of this study. **(A)** Flowchart of the Study; **(B)** Network Structure of DeepLabV3+; L, left; R, right; GLCM, gray level co-occurrence matrix; GLRLM, gray level run length matrix; GLSZM, gray level size zone matrix; First_order, first order statistics; NGTDM, neighboring gray tone difference matrix; GLDM, gray level dependence matrix. Conv: Convolution layer; Hospital A: The Second Affiliated Hospital of Zhejiang University School of Medicine, SAHZU; Hospital B: Tianjin Third Central Hospital, THTCH; Hospital C: The People’s Hospital of Yingshang, PHYS.

The radiologists for ROI are at least three years in ultrasound diagnosis. Besides, the labelme software in python is used to draw ROIs. Then, collected images was transformed into JPG image format and imported into PyCharm software. Third, the radiomics data were extracted using the Python package PyRadiomics (13) and 1682 radiomics variables from each side of the kidney (841 parenchyma and 841 sinus) were extracted in this study. Moreover, we calculated the intraclass correlation coefficient (ICC) between the extracted data from the manually and automatically delineated ROIs. The ICC values of variables that were higher than 0.7 were selected for further model building. Least absolute shrinkage and selection operator (LASSO) regression was used to select the significant features (14). Finally, the radiomics scores were calculated and the diagnostic performance was compared using the receiver operating characteristic (ROC) curve (**Figure 1A**).

### Deep Learning (DL) Algorithm

DeepLabV3+ improves pyramid-shaped hole pooling, cascaded multiple hole convolutions, and extensively uses batch normalization. First, DeepLabV3+ uses an atrous spatial pyramid pooling structure to mine multiscale contextual content information. The decoding structure gradually reconstructs the spatial information to capture object boundaries more effectively. Second, a new decoding module was added to reconstruct the boundary information. Third, we attempted to use MobileNet module as the backbone of the network to reduce the number of parameters and increase the speed of the network. The network structure of DeepLabV3+ is shown in **Figure 1B**.

### Data Enhancement

In the training set for DeepLabV3+, we first used the data enhancement strategies to extensively use ultrasound images, such as random horizontal flip, random scale change, and random Gaussian blur. After data enhancement, a five-fold increase in the number of pictures was achieved.

### Experimental Environment

The DeepLabV3+ network was built using PyTorch version 1.9.0 with Compute Unified Device Architecture (CUDA) version 11.1 (15). NVIDIA GeForce RTX3070 Ti platform was used in a Windows 10 operating system. Statistical modeling (LASSO) was performed using R and RStudio.

### Statistical Analysis

Continuous data with normal distribution are shown as mean ± standard deviation, and data with a non-normal distribution are shown as median (quartile interval). Categorical data are expressed as a number (percentage). The distributions of our data were measured using the Shapiro-Wilk test. In univariate analysis, continuous data were compared using Student’s t-test, one-way analysis of variance, Mann–Whitney U test, or Kruskal–Wallis H test, and categorical variables were compared using the χ^2^ test. Multiple comparisons were performed using Tukey correction.

## Results

### Baseline Characteristics Among the Three Medical Centers

A total of 499 patients were enrolled in this study: 131 T2DM patients and 53, 91, 18 patients with DKD stages II, III, IV, respectively, from SAHZU; 63 T2DM patients and 25, 31 patients with DKD stages II, III, respectively, from TJTCH; 52 T2DM patients and 13, 20, 2 patients with DKD stages II, III, IV, respectively, from PHYS.

The average ages were 57 (51–64), 59 (48-65), 57 (46-69) years for T2DM patients and 60 (51-68), 63 (51-75), 60 (49-70) years for DKD patients among the three datasets, respectively. Moreover, the percentages of male patients were 61.8, 57.1, and 25.0% among the T2DM patients and 59.3, 51.8, and 42.9% among the DKD patients in the three datasets, respectively.

In addition, among the three datasets, the average fasting blood glucose levels were 8.8 (6.4-12.2), 7.46 (5.72-9.99), 9.7 (7.0-12.0) mmol/L in T2DM patients, 7.4 (5.6-10.1), 8.8 (5.6-12.1), 9.4 (7.4-11.9) mmol/L in DKD patients, respectively. Further, among the three datasets, the eGFRs were 105.8 (94.8-115.8), 104.7 (85.7-123.8),100.2 (84.2-107.8) ml/min/1.73m^2^ in T2DM patients, 69.0 (33.3-105.3), 83.9 (47.0-99.1), 83.4 (47.9-100.4) ml/min/1.73m^2^ in DKD patients, respectively (**Table 1**).

**Table 1:** The Basic Characteristics of Study Patients in Three Medical Centers. SAHZU.

Variables	SAHZU		TJTCH		PHYS		*P*
T2DM	131 (44.7%)		63 (52.9%)		52 (59.8%)		**<** **0.05**
DKD Stage II	53 (18.1%)		25 (21.0%)		13 (14.9%)		
DKD Stage III	91 (31.1%)		31 (26.1%)		20 (23.0%)		
DKD Stage IV	18 (6.1%)		0 (0.0%)		2 (2.3%)		
**Demographics:**	**T2DM**	**DKD**	**T2DM**	**DKD**	**T2DM**	**DKD**	***P***
Age	57 (51-64)	60 (51-68)	59 (48-65)	64 (53-75)	57 (46-69)	61 (50-72)	P_1_^#^ P_2_^*^
Male (%)	81 (61.8%)	96 (59.3%)	36 (57.1%)	29 (51.8%)	13 (25.0%)	15 (42.9%)	P_1_^*^ P_2_^#^
BMI	24.5 (22.6-26.1)	25.3 (23.4-27.2)	25.9 (22.4-29.3)	26.1 (22.8-29.4)	25.6 (22.2-29.0)	25.7 (22.2-29.2)	P_1_^*^ P_2_^#^
Hypertension	56 (42.7%)	130 (80.2%)	36 (57.1%)	41 (73.2%)	20 (38.5%)	18 (51.4%)	P_1_^#^ P_2_^*^
DM duration	3285 (1825-5475)	3650 (2555-7118)	2555 (1095-4745)	3650 (1095-6388)	2920 (1095-3650)	3650 (1060-5475)	**P_1_^#^ P_2_^#^**
**Biochemical value**	**T2DM**	**DKD**	**T2DM**	**DKD**	**T2DM**	**DKD**	
HbA1c	8.9 (7.8-10.3)	8.4 (7.0-10.1)	8.3 (7.3-10.5)	9.1 (7.0-11.2)	9.7 (7.6-11.8)	8.8 (6.7-10.9)	P_1_^*^ P_2_^#^
FBG	8.8 (6.4-12.2)	7.4 (5.6-10.1)	7.5 (5.7-10.0)	8.6 (5.8-11.4)	9.7 (7.0-12.0)	9.3 (7.4-10.5)	P_1_^*^ P_2_^#^
Urea nitrogen	4.3 (5.1-6.2)	8.2 (5.2-12.2)	4.7 (4.0-5.4)	6.5 (4.7-8.5)	5.7 (4.4-7.1)	7.2 (4.9-10.5)	P_1_^*^ P_2_^*^
Creatinine	59.5 (47-71)	94 (60.5-200)	62 (55.5-70.5)	76 (59-113)	59.2 (47.9-70.6)	75 (61-110)	**P_1_^#^ P_2_^#^**
Uric acid	300 (257-350)	382 (307-449)	281 (229-331)	319 (200-437)	241 (161-322)	288 (236-364)	P_1_^*^ P_2_^*^
ACR	13.5 (8.9-18.0)	429.0 (58.0-2653)	5.6 (3.8-13.1)	244.9 (72.1-1166.4)	10.6 (4.3-17.0)	411.1 (57.0-2070.3)	P_1_^*^ P_2_^#^
eGFR	105.8 (94.8-115.8)	69.0 (33.3-105.3)	104.7 (85.7-123.8)	83.9 (47.0-99.1)	100.2 (84.2-116.2)	83.4 (47.9-100.4)	P_1_^*^ P_2_^#^

^*^: P-value ≤ 0.05; #: P-value > 0.05; P_1_: P-value of three datasets in T2DM groups; P_2_: P-value of three datasets in DKD groups; SAHZU, The Second Affiliated Hospital of Zhejiang University School of Medicine; TJTCH, Tianjin Third Central Hospital; PHYS, The People’s Hospital of Yingshang; T2DM, type 2 diabetes mellitus; DKD, Diabetic Kidney Disease; BMI, body mass index; DM, diabetes mellitus; HbA1c, glycated hemoglobin A1c; FBG, fasting blood-glucose; ACR, Albumin-to-Creatinine Ratio; eGFR, estimated glomerular filtration rat.

### DL-Based Anatomical-Level Segmentation

**Figure 2:**
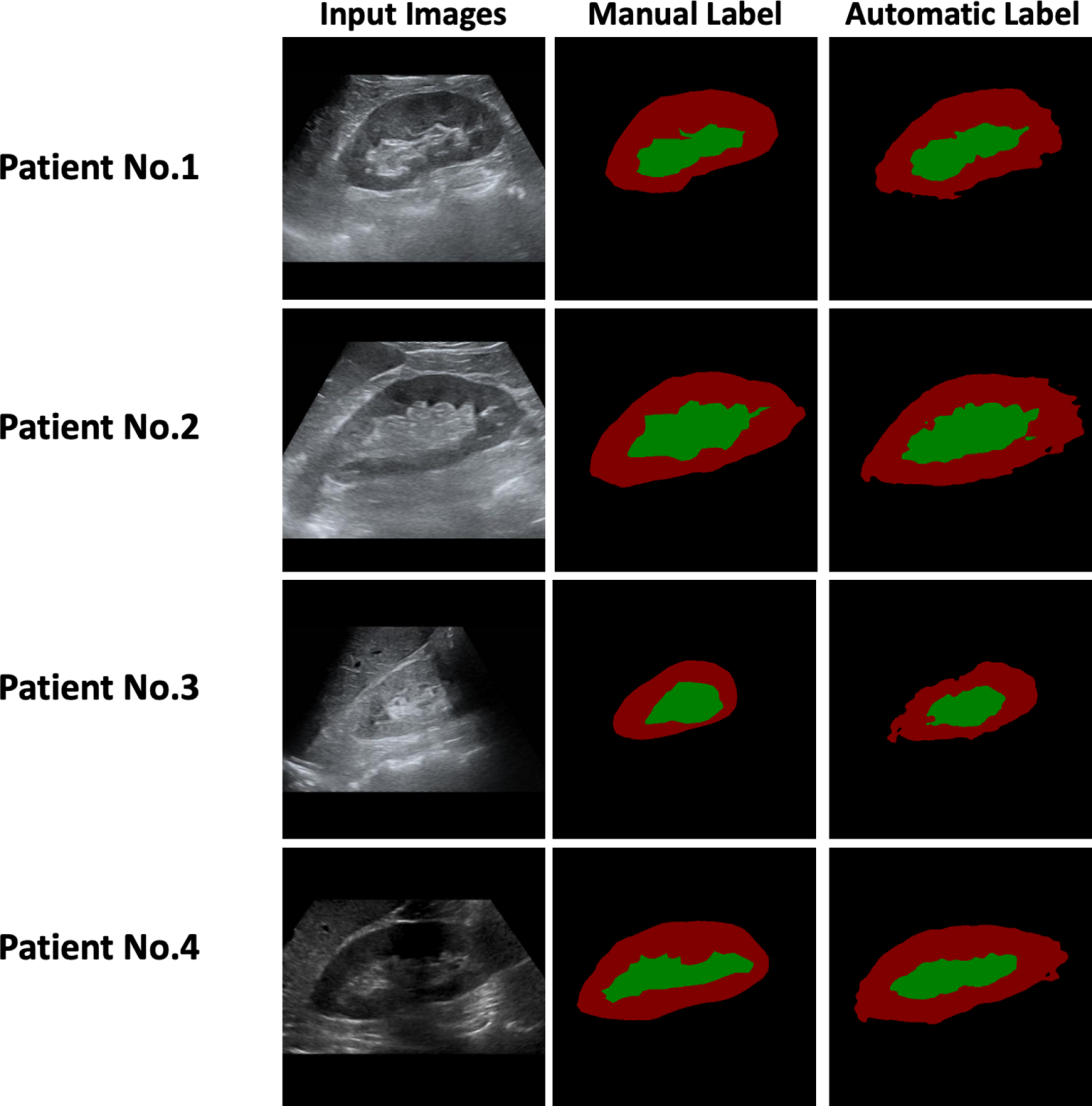
Manual and Automatic Segmentation using Ultrasound Images of the Patients.

Two radiologists independently delineated the kidney border, renal parenchyma, and renal sinus in ultrasound images, and the inconsistency was resolved through discussions. The DeepLabV3+ network was applied as an automatic anatomical-level segmentation technology, whose structure is illustrated in **Figure 1B**. As shown in **Figure 2**, the trained DeepLabV3+ model showed good segmentation ability in patients with clear ultrasound images (Patient Nos. 1 and 2). To further verify the robustness and accuracy of DL technology, we tested the model using the ultrasound images of patients with inferior ultrasound images (Patient Nos. 3 and 4). The trained model showed that it could compensate for the missing border caused by inferior ultrasound windows and maintain high segmentation accuracy.

**Figure 3:**
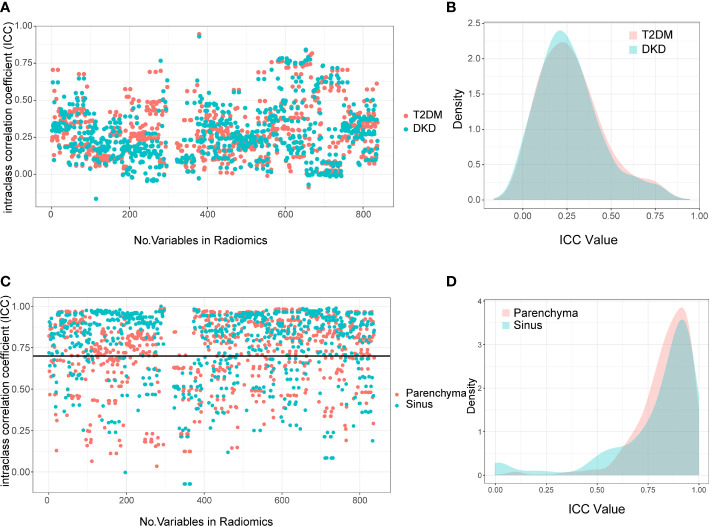
Interclass Correlation Coefficients and Density Plots between Extracted Radiomics Variables. The Interclass Correlation Coefficients plot **(A)** and Density Plots **(B)** between parenchyma and sinus in T2DM and DKD group. The Interclass Correlation Coefficients plot **(C)** and Density Plots **(D)** between manual and automatic ROI drawing methods in T2DM and DKD group.

Moreover, we verified the segmentation ability of DeepLabV3+ on a separate test set (N=50) from SAHZU and two independent test datasets from TJTCH and PHYS. From **Figure 3**, **Table 2**, it can be observed that the mean intersection-over-union (Miou) and mean pixel accuracy (mPA) of the SAHZU test set were 0.812 ± 0.003 and 0.890 ± 0.004, respectively. Moreover, the Miou and mPA of the TJTCH dataset were 0.781 ± 0.009 and 0.870 ± 0.002, respectively, and those of the PHYS dataset were 0.805 ± 0.020 and 0.893 ± 0.007, respectively (**Table 2**). These results demonstrate that high robustness and accuracy can be achieved by DL-based technology, thus providing a faster method for delineating the ROI.

**Table 2:** Mean Intersection-over-union and Mean Pixel Accuracy in Three Medical Centers.

Dataset	Miou	mPA
SAHZU	0.812 ± 0.003	0.890 ± 0.004
TJTCH	0.781 ± 0.009	0.870 ± 0.002
PHYS	0.805 ± 0.020	0.893 ± 0.007

Miou, mean intersection-over-union; mPA, mean pixel accuracy; SAHZU, Second Affiliated Hospital of Zhejiang University School of Medicine; TJTCH, Tianjin Third Central Hospital; PHYS, People’s Hospital of Yingshang.

### Correlation Between Extracted Radiomics Variables

In total, 3364 radiomics variables were extracted per patient, including 841 from the renal parenchyma and 841 from the renal sinus per kidney.

First, we calculated the correlation between the extracted variables from the parenchyma and sinus. The extracted variables were completely irrelevant, with ICC values of 0.236 (0.142-0.356) and 0.249 (0.133-0.374) in T2DM and DKD patients, respectively (**Figures 3A, B**). These results demonstrate that different parts of the kidney can provide different information.

Next, we calculated the ICC between radiomics variables extracted using manual and DL-based automatic methods to select robust variables for further analysis. As expected, the variables extracted using manual and automatic methods were highly correlated. Moreover, the median and interquartile range of ICC in the parenchyma and sinus were 0.871 (0.728-0.937) and 0.860 (0.779-0.927), respectively (**Figures 3C, D**). The radiomics variables with ICC > 0.7 were selected in this study; thus, 2066 radiomics variables, including 974 and 1092 variables extracted from parenchyma and sinus, respectively, were used in the model building step.

### Utilization of Radiomics Variables From Parenchyma and Sinus for Stratifying DKD Patients

**Figure 4:**
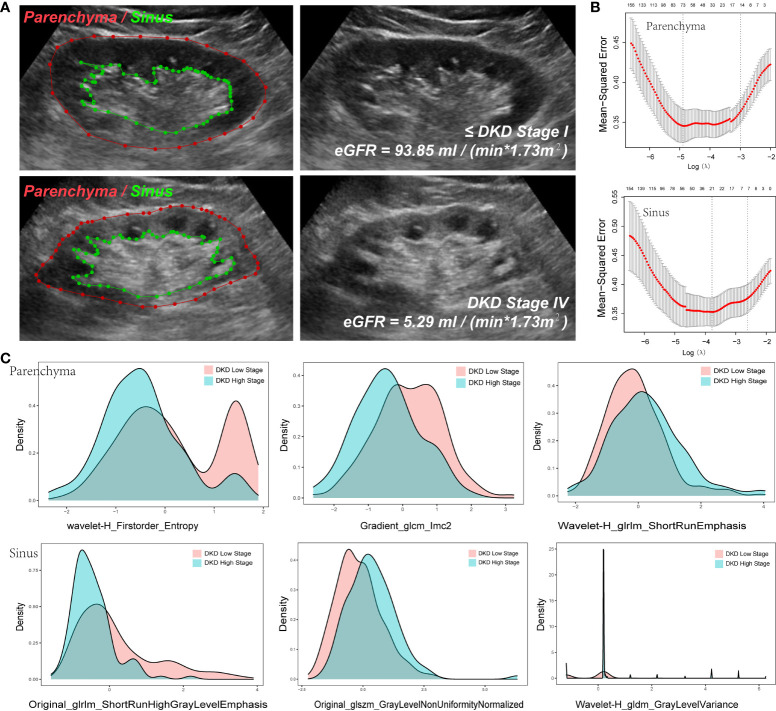
Variables extracted from Parenchyma and Sinus. **(A)** The ROI of parenchyma and sinus in two DKD stage patients. **(B)** The Mean-square error plot of LASSO regression in parenchyma and sinus model. **(C)** The Density Plots between Extracted Radiomics Variables in parenchyma and sinus.

The kidneys of patients with a high DKD stage tend to show higher echogenicity in both the parenchyma and sinus (**Figure 4A**), which can provide evidence that the radiomics variables have the potential to stratify DKD patients.

To reduce the dimensions of the variables, the LASSO procedure was performed. A total of 94 variables were selected, including 24 from the feature class of gray level co-occurrence matrix, 12 from gray level run length matrix, 12 from gray level size zone matrix, 16 from first order statistics, 3 from neighboring gray tone difference matrix, 6 from gray level dependence matrix, and 21 from wavelet decompositions (**Figure 4B**, **Table 3**).

**Table 3:** Class of Extracted variables.

Variables Class	Sinus	Parenchyma
GLCM	7	17
GLRLM	2	10
GLSZM	3	9
First_order	5	11
NGTDM	1	2
GLDM	0	6
Wavelet	3	18

GLCM, gray level co-occurrence matrix; GLRLM, gray level run length matrix; GLSZM, gray level size zone matrix; First_order, first order statistics; NGTDM, neighboring gray tone difference matrix; GLDM, gray level dependence matrix.

Note that 73 variables from parenchyma and 21 from sinus were selected (**Figure 4B**), as illustrated in the density plot (**Figure 4C**), and the value distribution of wavelet-H_Firstorder_Entropy (WHFE) and gradient_glcm_lmc2 (GGL) from the parenchyma were shifted to the left in the high DKD stage, whereas the value of wavelet-H_glrlm_shortRunEmphasis (WHGS) was shifted to the right in the high DKD stage. Moreover, the value of glrlm_shortRunHighGrayLevelEmphasis (GSHGLE) was shifted to the left in the high DKD stage, whereas the value of glszm_GrayLevelNonUniformyNormalized (GGLNUN) was shifted to the right in the high DKD stage. Further, the distribution of the value of wavelet-H_gldm_GrayLevelVariance (WHGGLV) in the high DKD stage was more spiculate than in the low DKD stage.

These results demonstrated that the variables extracted from both the parenchyma and sinus could provide positive diagnostic value in the diagnosis and follow-up of DKD, particularly in the low- and high-stage DKD (**Figure 4**).

### Diagnostic Performance of Ultrasound-Based Radiomics in Stratifying DKD Patients

We randomly separated the data of the 499 patients from the three medical centers into three parts: 424 (85%) patients were divided into training and validation datasets and 75 (15%) patients were divided into independent test datasets. In the model building step, a k-fold cross-validation method was applied to the training and validation datasets to calculate the area under the curve (AUC), and the differences between the groups were compared. After the previous step, the best model was tested using independent test datasets, and the ROC and AUC were plotted and calculated.

**Figure 5:**
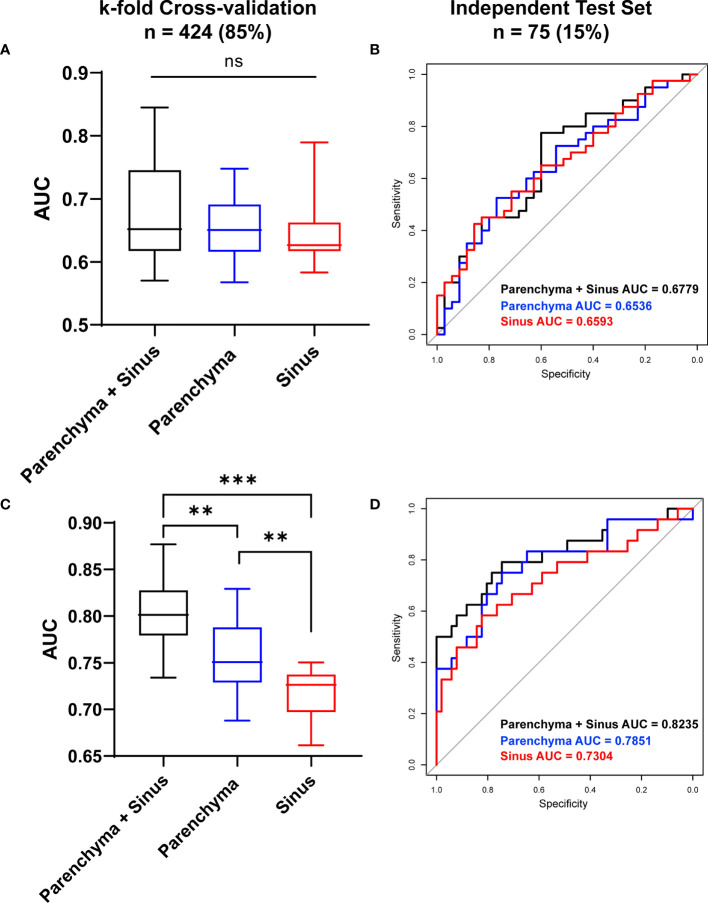
Diagnostic Performance of ultrasound-based radiomics to stratify DKD patients. The diagnostic performance to differentiate DKD and T2DM patient in cross-validation datasets **(A)** and in independent test set **(B)**. The diagnostic performance to differentiate high (≥ stage III) and low (≤ stage II) DKD stages in Cross-validation datasets **(C)** and in independent test set **(D)**. AUC, Area under curve; *P < 0.05, **P < 0.01, ***P < 0.001; ns, no significance.

While differentiating between DKD and T2DM patients, the radiomics model achieved moderate diagnostic performance with AUCs of 0.674 ± 0.074 in the parenchyma + sinus model, 0.6561 ± 0.0537 in the parenchyma model, and 0.6457 ± 0.0514 in the sinus model (**Figure 5A**). No statistical differences were found among the three models (all *P* > 0.05). In the independent test set, the AUCs of parenchyma + sinus, parenchyma, and sinus were 0.6779, 0.6536, and 0.6593, respectively (**Figure 5B**).

When differentiating between the high (≥ stage III) and low (≤ stage II) DKD stages, the radiomics model that combined the information from parenchyma and sinus achieved the highest diagnostic performance with AUC = 0.803 ± 0.037 (all *P*<0.05) after k-fold validation. Moreover, the AUCs of the radiomics model using only parenchyma and sinus variables were 0.75695 ± 0.038 and 0.716 ± 0.026, respectively (**Figure 5C**). In the independent test set, the AUCs of the models using parenchyma + sinus, parenchyma, and sinus variables were 0.8235, 0.7851, and 0.7304, respectively (**Figure 5D**).

In this study, the T2DM patients were a mix of T2DM patients without kidney function disorders and DKD stage I patients. These results demonstrated that the ultrasound images of patients in the early stage of DKD are similar to those of T2DM patients, which results in a moderate diagnostic performance of the radiomics model. However, the ultrasound-based radiomics model demonstrates good potential in differentiating between the low and high DKD stages, which is more useful for the stratification of patients with DKD.

### Diagnostic Performance of DL-Based Automatic Segmentation, Radiomics for DKD

To further study the performance of the DL-based automatic segmentation, radiomics model in identifying DKD patients, we calculated and compared the AUC between the manual and automatic methods.

**Figure 6:**
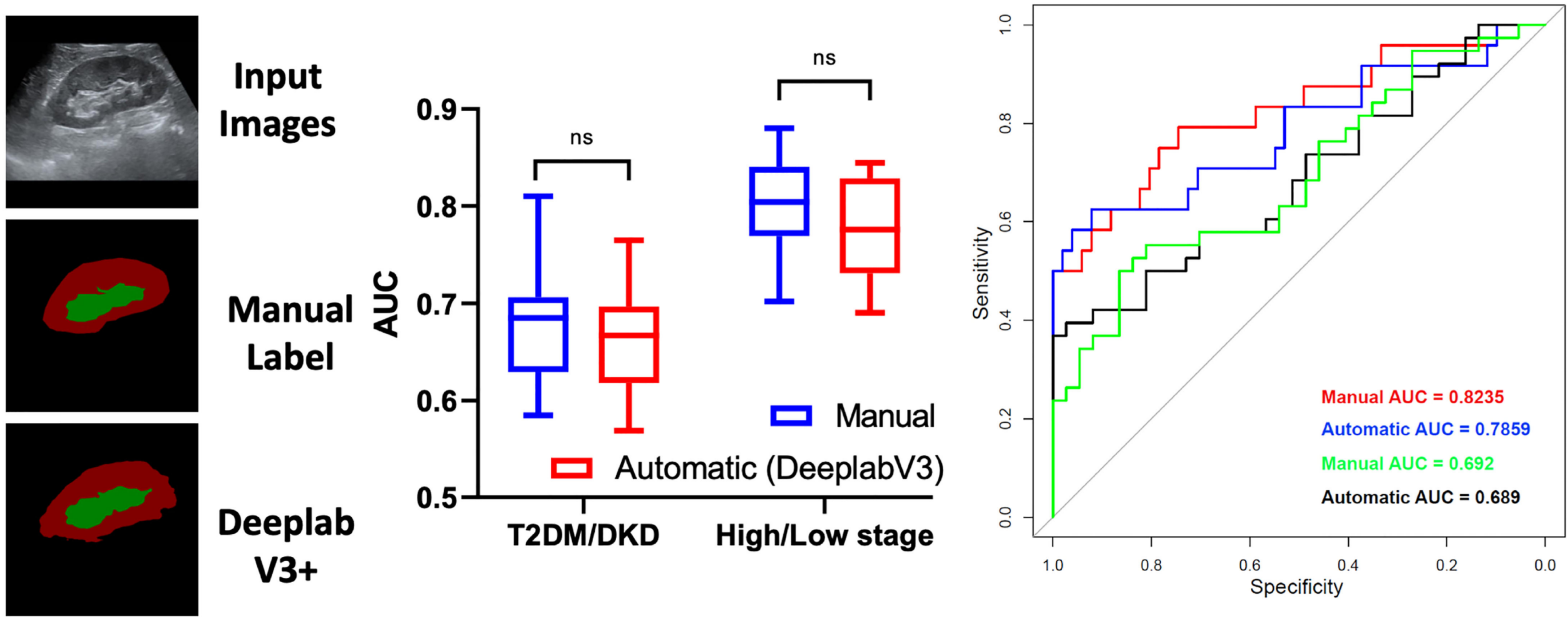
Diagnostic Performance of deep learning-based automatic segmentation, radiomics for diabetic kidney disease. ns, no significance.

After k-fold cross-validation, the AUCs of the manual and automatic methods when differentiating between DKD and T2DM patients were 0.6797 ± 0.058 and 0.6626 ± 0.0547, respectively. Moreover, the AUCs of the manual and automatic methods of differentiating between patients at high and low DKD stages were 0.7967 ± 0.054 and 0.7732 ± 0.05478, respectively. There was no statistically significant difference between the AUCs of the manual and automatic methods (all *P <*0.05) Further, the AUCs of T2DM/DKD and high/low DKD stage while using manual and automatic methods were 0.692, 0.689, 0.8235, 0.7859, respectively, on the independent test set (**Figure 6**). The results demonstrated that manual and automatic segmentation, radiomics models achieved similar diagnostic performance.

## Discussion

Ultrasonography is an ideal evaluation tool and is widely used for the identification and analysis of several diseases (16). Although, researchers can identify abnormal renal echogenicity, renal size, and other features in the diagnosis of kidney dysfunction through ultrasonography (17–19), the interpretation of ultrasound images by the naked eye is subjective and certain high-dimensional information can be missed; these problems can be solved using radiomics technology. However, accurate anatomical segmentation of the ROI, which is a time-consuming and experience-dependent process, is vital for effective utilization of radiomics technology. Therefore, in this retrospective multicenter study, we developed a DL-based automatic segmentation, radiomics technology to evaluate the diagnostic performance of radiomics technology for DKD patients and evaluated its potential for clinical application.

Moreover, in underdeveloped regions or primary clinics, it may be costly and challenging to train or recruit experienced doctors to fulfill the large medical demand. One of the solutions is fifth generation communication technology, which can achieve remote medical systems by connecting experienced doctors with patients online. In addition, the DL-based automatic segmentation, radiomics technology can perform the role of an intelligent machine doctor, which is portable if configured using a handheld ultrasound device and can quickly and effectively determine the results. This work demonstrates significant potential for achieving automatic labor-free diagnosis and follow-up of DKD, and is our further research focus.

However, this study has certain limitations. First, the retrospective nature of the study may have influenced the accuracy of the diagnostic performance to a certain extent. Second, the heterogeneity among the three medical centers may affect the degree of accuracy. Third, patients with stage I DKD were unavailable in this study. The clinical definition of these patients is primarily defined by pathologic findings that are difficult to access in clinical practice. This resulted in a mixture of T2DM patients without kidney function injury and DKD stage I patients in the dataset of T2DM participants.

In summary, we first verified the robustness of the automatic segmentation method using the DeepLabV3+ network. This result is supported by the research work of Yin et al. (20) the DL-based classification network can achieve good segmentation of the kidney. Moreover, we identified that the renal parenchyma and sinus can provide different information to support the classification model. Accurate anatomical-level segmentation was achieved in this study. The automatic segmentation network achieved superior performance in the segmentation of ultrasonography images with both good and bad ultrasound windows. The Miou and mPA of the automatic segmentation method were high for the independent test set and the datasets from the other independent medical centers. In fact, the DL-based segmentation could reduce the time for hand annotation from 1 h to lower than a few seconds for 100 images, which significantly reduces labor costs. Second, we demonstrated that the ultrasound-based radiomics model achieves a high diagnostic value when differentiating between different DKD stages and has the potential to stratify patients with DKD. The diagnostic performance of artificial intelligence (AI) technology has been supported by the work of Sudharson et al. (21) for certain kidney disorders. Moreover, the work of Chin-Chi Kuo et al. also supported our result (22) in which the authors reported that the prediction of the eGFR and accuracy (85.6%) by an AI-based model was higher than that by experienced nephrologists (60.3-80.1%). The AUC of ROC in our study was 0.803 ± 0.037, which is marginally lower than that reported by Chin-Chi Kuo et al. This may be due to the different clinical definitions of DKD and CKD and the relatively smaller number of patients with severe kidney dysfunctions (eGFR < 30 mL/min/1.73 m^2^) in our study (22). Li et al. reported that 3D ultrasound also has potential value in the diagnosis of diabetic nephropathy (DN) and may act as an auxiliary diagnosis for DN (23), which suggests that 3D ultrasound radiomics can be considered in future studies.

## Conclusion

In this study, we developed a DL-based automatic segmentation, radiomics technology to stratify DKD patients, which could reduce the time for hand annotation from few hours to less than a few seconds for 100 images and could achieve satisfactory diagnostic performance in the diagnosis and follow-up of DKD patients.

## Data Availability Statement

The original contributions presented in the study are included in the article/**Supplementary Material**. Further inquiries can be directed to the corresponding authors.

## Ethics Statement

This study involving human participants was reviewed and approved by the ethics committee of The Second Affiliated Hospital of Zhejiang University School of Medicine. Written informed consent for participation was not required for this study in accordance with the national legislation and institutional requirements.

## Author Contributions

PH, XJ, and QC designed the study, participated in the supervision and coordination of the study; JC, PJ, YS, and LF conceived and designed the study; JL, HC, LX, FQ, ZC, JS, YaZ, WX, CC, YanZ, JY, and CZ collect the data; JC, PJ, YS, and LF analyzed the data; PH, XJ, QC, JC, PJ, YS, and LF contributed to the writing, review and revision of the manuscript. All authors read and approved the final manuscript.

## Funding

This work was supported by the National Natural Science Foundation of China (NO. 82030048, 82102191, 82001818, and 82102052), Key Research and Development Program of Zhejiang Province (NO. 2019C03077), Natural Science Foundation of Zhejiang Province (NO. LQ20H180009, Y16H180019, LQ19H180004 and LQ21H180007).

## Conflict of Interest

Author HC was employed by Hangzhou Supor South Ocean Pharmaceutical Co., Ltd.

The remaining authors declare that the research was conducted in the absence of any commercial or financial relationships that could be construed as a potential conflict of interest.

## Publisher’s Note

All claims expressed in this article are solely those of the authors and do not necessarily represent those of their affiliated organizations, or those of the publisher, the editors and the reviewers. Any product that may be evaluated in this article, or claim that may be made by its manufacturer, is not guaranteed or endorsed by the publisher.

## Abbreviations

DKD, diabetic kidney disease; DL, deep learning; DN, diabetic nephropathy; T2DM, type II diabetes mellitus; Miou, mean intersection-over-union; mPA, mean pixel accuracy; AUC, area under the curve; ROI, region of interest, CKD, chronic kidney disease; eGFR, estimated glomerular filtration rate, SAHZU, Second Affiliated Hospital of Zhejiang University School of Medicine; PHYS, People’s Hospital of Yingshang; TJTCH: Tianjin Third Central Hospital; UACR, urinary albumin-to-creatinine ratio; UAER, urinary albumin excretion rate; LASSO, Least absolute shrinkage and selection operator; ICC, intraclass correlation coefficient; CUDA, Compute Unified Device Architecture; BMI, body mass index; HbA1c, glycated hemoglobin A1c; FBG, fasting blood-glucose; ACR, Albumin-to-Creatinine Ratio; GLCM, gray-level co- occurrence matrix; GLRLM, gray-level run-length matrix; GLSZM, gray-level size zone matrix; GLDZM, gray-level distance zone matrix; NGTDM, neighborhood gray tone difference matrix; NGDLM, neighboring gray-level dependence matrix; ROC, receiver operating characteristic; WHFE, wavelet-H_Firstorder_Entropy; GGL, gradient_glcm_lmc2; WHGS, wavelet- H_glrlm_shortRunEmphasis; GSHGLE, glrlm_shortRunHighGrayLevelEmphasis; GGLNUN, glszm_GrayLevelNonUniformyNormalized; WHGGLV, wavelet-H_gldm_GrayLevelVariance.
