# Supplementary material for: Auto-Segmentation Ultrasound-Based Radiomics Technology to Stratify Patient With Diabetic Kidney Disease: A Multi-Center Retrospective Study
Source: Front Oncol. 2022 Jul 4;12:876967. doi: 10.3389/fonc.2022.876967 (PMC9290767; doi:10.3389/fonc.2022.876967)
Supplement: Supplementary file 1 [file Table_1.docx]

**The Define of Diabetic Kidney Disease**

The following parameters were used to define DKD: 1) urinary albumin-to-creatinine ratio (UACR) > 30 mg/24 h, with an increase greater than twice the original value in three subsequent examinations conducted over 3-6 months; 2) eGFR < 60 ml min^-1^ for more than three months; 3) pathological result of kidney biopsy shows evidence of DKD.

**Clinical Stage of Diabetic Kidney Disease**

DKD stage I, called as high-filtration stage, was defined as having normal or a marginally elevated eGFR (> 90 mL/min/1.73 m2) and negative microalbuminuria; DKD stage II, called as microalbumin stage, was defined as having a urinary albumin excretion rate (UAER) of approximately 20-200 μg/min or 30-300 mg/24 h and eGFR > 60 mL/min/1.73 m2; DKD stage III, called as massive albuminuria stage, was defined as having normal UACR >300 mg/g, UAER > 200 ug/min or >300 mg/24 h and eGFR > 15 mL/min/1.73 m2; DKD stage IV, called as renal failure stage, was defined as having eGFR < 15 mL/min/1.73 m2.

**Experimental Environment**

The DeepLabV3+ network was built using PyTorch version 1.9.0 with Compute Unified Device Architecture (CUDA) version 11.1 [1]. NVIDIA GeForce RTX3070 Ti platform was used in a Windows 10 operating system. Statistical modeling (LASSO) was performed using R and RStudio.

**Radiomics features:**

The readiomics features we extracted in our study is basing on the pyradiomics [2] document.

**Reference:**

1. Chen KM, Cofer EM, Zhou J et al. Selene: a PyTorch-based deep learning library for sequence data. Nat Methods 2019; 16: 315-318. doi:10.1038/s41592-019-0360-8

2. van Griethuysen JJM, Fedorov A, Parmar C et al. Computational Radiomics System to Decode the Radiographic Phenotype. Cancer Res 2017; 77: e104-e107. doi:10.1158/0008-5472.Can-17-0339
